# Future challenges in the *in vitro* and *in vivo* evaluation of biomaterial biocompatibility

**DOI:** 10.1093/rb/rbw001

**Published:** 2016-03-10

**Authors:** James M. Anderson

**Affiliations:** Case Western Reserve University, 2103 Cornell Road, WRB 5105, Cleveland, OH 44106-7288, USA

**Keywords:** biomaterials, nanomaterials, tissue engineering

## Abstract

As the science and engineering of biomaterials continues to expand with increased emphasis on the development of nanomaterials and tissue engineering scaffolds, emphasis also must be placed on appropriate and adequate approaches to the *in vivo* and *in vitro* evaluation of biomaterial biocompatibility/biological response evaluation. This article presents six topic areas that should be addressed by investigators as they move forward in the development of new systems for regenerative medicine. Most certainly, there are other areas that require attention to detail and a more complete understanding of the strengths and weaknesses of various experimental approaches to biomaterial/biological response evaluation. The small number of issues addressed in this article is only meant to bring to the attention of prospective investigators and authors, the importance of the development of adequate and appropriate evaluation techniques that address the unique features of biomaterials utilized in the development of new medical devices.

## Introduction

In the context of grand challenges for biomaterials in the 21st century, this article presents perspectives believed by the author to be important and significant in addressing *in vitro* and *in vivo* evaluation of biomaterial biocompatibility. The following topics are not meant to provide an in-depth perspective but rather to stimulate biomaterial scientists to consider these topics in the context of their specific materials including nanomaterials and tissue engineering scaffolds. As biomaterials science and engineering has progressed over the past some 70 years, the design and development of new biomaterials has shifted to the development of bioactive materials where the evaluation of biocompatibility becomes more specific and significant. Unfortunately, biocompatibility or biological response evaluation assessment has not developed at the same pace and thus the goals and aims of biological response evaluation require reconsideration and re-evaluation. The following presents the author’s views on a small number of these topics which should be taken into consideration in the biological response evaluation of new biomaterials including nanomaterials, tissue scaffolds and complex combination systems where cells and/or proteins may be a component of the biomaterial system.

## ISO 10993-1 revision

**Figure 1.**
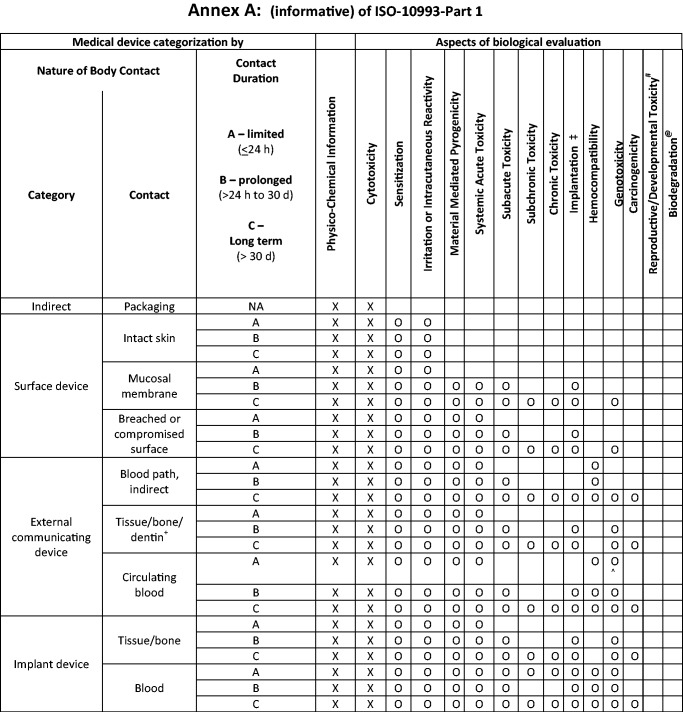
Considerations for biological assessment “X” indicates tests that must be performed; “0” indicates tests that should be considered.

ISO 10993-Part 1, Biological Evaluation of Medical Devices—Part 1: Evaluation and Testing within a Risk Management Process is currently undergoing revision. This international standard with its 20 parts is most commonly used for biocompatibility or biological response evaluation of biomaterials. Table 1 presents aspects of biological assessment that should be considered in the evaluation of new biomaterials or ‘old’ biomaterials used in new applications. The ISO 10993-1 standard is composed of two components: a normative component which requires all the aspects presented in Table 1 to be addressed. This does not necessarily mean that these tests have to be carried out but, rather, they should be considered by the biomaterial scientists and testing can then be determined based on the nature of body contact and contact duration. The second component of ISO 10993-1 is an informative component (Annex A) which provides suggestions and considerations for biological assessment of biomaterials. Figure 1 is a table which combines the aspects for biological assessment together with the nature of body contact and contact duration to assist the biomaterial scientists in the selection of appropriate and adequate evaluation. In the ISO 10993 standard, parts 2 to 20 provide details on the tests that should be considered. Not all tests may be required for a given biomaterial; in many cases, a written rationale for not carrying out testing may be provided.

**Table 1.** Aspects of biological assessment

Physicochemical information
Cytotoxicity
Sensitization
Irritation or intracutaneous reactivity
Material-mediated pyrogenicity
Systemic acute toxicity
Subacute toxicity
Subchronic toxicity
Chronic toxicity
Implantation
Hemocompatibility
Genotoxicity
Carcinogenicity
Reproductive/developmental toxicity
Biodegradation

It should be considered that Table 1 and Figure 1 coupled with a schematic (not presented) permits the biomaterial scientist to adequately and appropriately determine the selection of biological tests required for further evaluation of the device based on the chemical nature of the materials and the type, contact and duration of contact. Ultimately, a toxicological risk assessment must be carried out based on the findings of the biocompatibility tests or biological response evaluations. Table 1 and Figure 1 are draft documents which are currently being considered for inclusion in the normative and informative sections of ISO 10993-Part 1 and therefore provide guidance to the investigator. It is anticipated that the revision of ISO 10993-Part 1 will be formalized and completed by 2017.

## Cytotoxicity—cell lines

Cytotoxicity evaluation is required for virtually every medical device and its component biomaterials. Unfortunately, cytotoxicity testing most commonly uses fibroblasts *in vitro* for a short period of time, commonly up to 7 days, and then claims are usually made that the biomaterial is noncytotoxic. Rarely do biomaterial scientists consider that cell lines are tumor derived and therefore do not necessarily represent the specific cells and tissues that may come in contact with their specific medical device or biomaterial. In addition, data at single time points are usually presented and the kinetics over an extended time period is not considered. Rarely is lag time to proliferation, proliferation rate and appropriate controls included in the cytotoxicity analysis. The appropriate cell line for the intended application should be utilized for cytotoxicity evaluation.

For the past 35 years, the author’s research interests with biomaterials has focused on the foreign body reaction composed of macrophages and foreign body giant cells (fused macrophages), which are found at the tissue/implant interface. Our work has utilized primary cells, i.e., human blood–derived monocytes that are differentiated into macrophages and then utilized under various conditions for biocompatibility analysis. Table 2 provides a list of macrophage cell lines used in biomaterials studies. It is noteworthy that all these cell lines are tumor derived, produced from either leukemias or lymphomas and the majority are from rodent tumors. Rarely is information presented on the similarities and differences of these tumor-derived cell lines in comparison with human blood–derived monocytes and macrophages. A significant difference between the human blood–derived monocyte and the cell lines in Table 2 is that human blood–derived monocytes do not undergo replication but are an end-stage cell that does not proliferate, unlike the macrophage cell lines identified in Table 2.

**Table 2.** Macrophage cell lines used in biomaterial studies

Cell line	Source
HL-60	Human: promyelocytic leukemia
IC-21	Mouse: transformed peritoneal macrophages
J774	Mouse: histocytic lymphoma
J774A.1	Mouse: histocytic lymphoma
P388D1	Mouse: transformed lymphoma
RAW	Mouse: transformed lymphoma
RAW 264.7	Mouse: transformed lymphoma
THP-1	Human: acute monocytic lymphoma
U937	Human: histiocytic lymphoma
‘Macrophages’	Mouse: peritoneal surface

## Biomaterial carcinogenicity

Although biomaterial carcinogenicity is an important and potentially significant issue with new biomaterials, two aspects of biomaterial carcinogenicity are presented. In the 1950s and 1960s, Oppenheimer carried out extensive studies on the ability of implant materials to initiate tumors in rodents. Oppenheimer’s pioneering effort clearly identified the potential for tumor development in rodents. However, subsequent studies in tumors have not replicated the so-called ‘Oppenheimer effect’ which was identified in rodents. In 1955, Oppenheimer *et al*. [1] published a significant manuscript in which various commercial polymers in various forms were tested for tumor development. Table 3 presents these data and clearly identifies the significance of the form of the material and its potential to produce tumors in rodents. Our interpretation of these results is that the increased surface area in a given volume of biomaterial produces an increased foreign body reaction (macrophages and foreign body giant cells), which results in a decrease in tumorigenesis or carcinogenicity. These results may have significant implications for high surface area medical devices such as nanoparticles and tissue engineering scaffolds.

**Table 3.** Polymers as carcinogenic agents in rodents—effect of form

Malignant tumors			
Polymer	Film	Perforated film	Textile
Mylar/Dacron	19.5%	4.8%	0
Nylon 66	27.0%	6.5%	0
Polyethylene	20.0%	14.6%	2.5%
		Powder	0
Polyvinyl chloride	38.6%	0	–

Note. Increased surface area with increased foreign body reaction results in decreased tumorigenesis.

In the early 1990s, a polyurethane foam material was utilized as a covering for silicone rubber breast implants. The concept here was to disrupt the fibrous capsule that formed around breast implants and reduce the contracture of this fibrous capsule which was found in the clinical use of silicone rubber breast implants. The polyurethane-covered breast implants utilized Scotfoam, which was a reticulated foam/sponge that utilized 2,4-toluenediisocyanate and 2,6-toluenediisocyanate as hard segments for the polyurethane foam and poly(diethylene glycol adipate), a 3000 molecular weight polyoxypropylene polyether polyol as the soft segment of the segmented polyurethane. The adipate ester bond within the soft segment was conducive to cleavage leading to biodegradation/bioresorption and the release of 2,4-toluenediamine (TDA), a known carcinogen, was considered to increase the potential for carcinogenicity. However, although the polyurethane-covered breast implants underwent biodegradation/bioresorption as identified in human clinical studies, the risk of carcinogenicity was no more than one in ∼1 million cases. Interestingly, no detectable 2,4-TDA was found in the blood serum of patients receiving this polyurethane-coated breast implant and urine analysis demonstrated only small amounts of free TDA [2–4]. However, when the urine of these patients was hydrolyzed in 6 M hydrochloric acid at 110°C for 1 h, free TDA was enhanced. These findings demonstrate that the urinary clearance of the 2,4-TDA still contained bound polyether components and the great majority of the polyurethane bonds between the soft and hard segments were not cleaved to produce TDA. These studies point out the importance of determining the biodegradation/bioresorption products when biodegradable/bioresorbable biomaterials are utilized in a medical device.

## Biocompatibility ‘hot buttons’

Biocompatibility hot buttons are terms commonly utilized in regulatory submissions for presentation in reports to regulatory agencies. These terms usually are used by individuals who are not knowledgeable in the commonly accepted ‘tissue response continuum’ and invariably present the wrong impression in attempting to provide biocompatibility information. These biocompatibility hot buttons include chronic inflammation, immune response, innate immunity and acquired immunity. The tissue response continuum is the time-dependent changes that occur following implantation of medical devices and biomaterials. They include acute inflammation characterized by the presence of polymorphonuclear leukocytes (neutrophils); chronic inflammation characterized by the presence of monocytes and lymphocytes; foreign body reaction characterized by the presence of macrophages and foreign body giant cells (fused macrophages) at the tissue/implant interface; granulation tissue characterized by initiation of the healing response that includes the presence of fibroblasts and new blood vessels and, finally, fibrous encapsulation characterized by fibroblasts and collagen. Unfortunately, the foreign body reaction is commonly but incorrectly identified as chronic inflammation and the tissue response continuum clearly points out the significant difference in cell types that are present in these two distinct responses. Thus, the use of chronic inflammation to describe the foreign body reaction in regulatory submissions is inappropriate and raises concern regarding the biocompatibility of the given medical device or biomaterial.

Authors of published manuscripts commonly use the term ‘immune response’ without being specific as to whether or not this response is an ‘innate’ or ‘acquired or adaptive’ response. The innate immune response is actually the early and resolving inflammatory response seen within the first 2 weeks following implantation of a biocompatible medical device or biomaterial. The use of the terminology for inflammation is a much better way to present or identify this response than is ‘innate immunity’. As noted, chronic inflammation is characterized by monocytes and lymphocytes and it is these cells that can lead to a specific or acquired immune response. The most common types of acquired immune responses are Type I immune response which is a hypersensitivity reaction and Type IV immune response which is a cell-mediated hypersensitivity reaction involving antigen–antibody interactions and activated ‘T lymphocytes’. Type IV adaptive or acquired immune responses have been seen with certain types of orthopedic devices including total artificial hip joint prostheses and fracture fixation plates. These are wear particulate and the release of cobalt and chromium ions over the course of the implant time.

To avoid confusion and misinterpretation, it is recommended that the terminology used to describe inflammation and immune responses should also include a clear definition of the specific terminology being used. Inclusion of specific definitions with identification of cell types involved in the responses is most helpful in clearly identifying the cellular response to implanted medical devices and biomaterials over the tissue response continuum.

## Issues in biological response evaluation

Over the course of the author’s 45 years of research experience with medical devices and biomaterials, as well as his editorship of the ‘Journal of Biomedical Materials Research-Part A’, the author has identified a number of significant and important issues that are rarely addressed in published manuscripts attempting to provide information on biological response evaluation.

First, the experimental design for biocompatibility testing must be based on the unique properties of the biomaterial and its intended use, i.e., medical devices, prosthesis and so on. It is generally known that a biomaterial may be biocompatible in one application but not in another. Therefore, experimental design becomes important in attempting to determine biocompatibility and the intended or expected biological response.

Second, the experimental design must utilize quantitative assays and statistical analysis when possible. Third, the utilization of cell lines for cytotoxicity testing and *in vitro* biocompatibility testing must be clearly identified and appropriate for the intended use of the medical device, prosthesis or biomaterial.

As the science of biomaterials has progressed, authors have attempted to identify molecular mechanisms responsible for the tissue response and the tissue/cellular biological response. It is common now to utilize *in vivo* ‘knock-out’ systems to identify molecular mechanisms. However, the question remains what is being ‘knocked out’ in these respective animal models? Rarely is information provided as to the other types of reactions that may be either enhanced or reduced in the use of the so-called specific knock-out animal models. Nature provides multiple pathways for achieving the same response and knock-out models may not fully appreciate the duality that may exist in the initiation or reduction of certain biological responses. An example of this has been presented in the author’s work where it was found that utilizing Interleukin-4 (IL-4) knock-out mice still resulted in the fusion of macrophages to form foreign body giant cells at the tissue/material interface. It was subsequently discovered that even though IL-4 was knocked out, IL-13 was responsible for facilitating macrophage fusion to form foreign body giant cells. It was later determined in the author’s laboratory that the IL-4 receptor on macrophages also could bind IL-13, which led to macrophage fusion and foreign body giant cell formation [5–11].

Finally, and perhaps most importantly, the *in vivo* validation of *in vitro* results must be carried out. It is common in the literature to make exaggerated claims based on *in vitro* results and no follow-up with *in vivo* studies is presented to verify these claims and hypotheses. *In vivo* validation of *in vitro* results must be carried out if the proposed biomaterial is to be utilized in a medical device or prosthesis.

## Future challenges for biocompatibility/biological response evaluation

Table 4 presents a short and incomplete list of the future challenges for biocompatibility/biological response evaluation. Efforts to bring new biomaterials, medical devices and prostheses to clinical use and application must address these challenges. As outlined in ISO 10993, the *in vivo* verification of *in vitro* results is necessary with a perspective on the unique applications of the biomaterials in the unique medical device under consideration.

**Table 4.** Future challenges for biocompatiblity/biological response evaluation

In vivo verification of in vitro results
New biological response tests
Nanodevices
Tissue engineered scaffolds
Materials, cells and proteins
Phenotypic interactions
IL-1–IL-1ra
FGF–MMP–TIMP
Fibrous capsule remodeling
Fibrous capsule diffusibility
Pharmacokinetics

New biological response tests must be devised to appropriately and adequately identify biological responses to nanodevices and tissue engineered scaffolds which may contain various components such as new materials, cells and proteins. This is a significant challenge and it is clear that new and innovative biological response test systems must be developed to adequately and appropriately test nanodevices and tissue engineered scaffolds.

Phenotypic interactions must be considered in the biological response evaluation. Cytokine analysis now commonly is being used to identify the activity of various cell types in the biological response evaluation but, in many cases, this type of analysis falls short of providing a complete picture or interpretation of the response. Examples of this include the cellular production of IL-1. When macrophages and other cell types produce IL-1, the receptor antagonist for IL-1, IL-1ra, also is produced and can bind and inactivate the biological response of IL-1. Therefore, a more complete analysis and interpretation can be derived when both IL-1 and IL-1ra are analyzed [12]. A similar example involves the identification and presence of fibroblast growth factor (FGF). FGF produces matrix metalloproteinases (MMPs) and also produces the tissue inhibitor for matrix metalloproteinases (TIMP). Thus, a more complete analysis would include the quantitation of both MMP and TIMP when attempting to determine the significance of the release and presence of FGF [13]. This is important in fibrous capsule remodeling which is now being considered as important in the tissue response to various medical devices and biomaterials. In considering various types of injected or implanted controlled release systems, the diffusibility of the fibrous capsule may play a significant role in determining the pharmacokinetics and the pharmacodynamics of a given active agent. Earlier considerations of the fibrous capsule as being a barrier to the diffusion of higher molecular weight active agents may be incorrect as recent studies have shown that proteins on the order of 4000 molecular weight can provide adequate blood levels for their intended response [14]. Thus, one must not necessarily consider the fibrous capsule as being a diffusion barrier for active agents released from controlled release systems.

## Conclusion

The following are conclusions for the six issues discussed in this article. It should be understood that ISO 10993 is a living document and constantly evolving, dependent on the unique features of the materials considered for new medical device development in regenerative medicine. Utilization of appropriate cell lines for cytotoxicity has increased impact when cell lines similar to the tissues in implant sites are used. Regarding carcinogenicity, it must be understood that a complete and thorough literature search is necessary when considering this topic. The example given in this article is the Oppenheimer reference which is 60 years old, but still pertinent to today’s topics in biomaterial biocompatibility. In addition, the *in vitro* products from degradable systems should be thoroughly investigated in the *in vivo* context and not necessarily assumed from the given chemistry of any biomaterial construct. Biocompatibility hot buttons emphasize the importance of including definitions when citing different types of tissue responses. Issues in biological response evaluation and future challenges identify topics that must be considered in the early stages of biomaterial development of new complex systems for regenerative medicine and tissue engineering.

*Conflict of interest statement*. None declared.
